# Review of Alternative Solvents for Green Extraction of Food and Natural Products: Panorama, Principles, Applications and Prospects

**DOI:** 10.3390/molecules24163007

**Published:** 2019-08-19

**Authors:** Farid Chemat, Maryline Abert Vian, Harish Karthikeyan Ravi, Boutheina Khadhraoui, Soukaina Hilali, Sandrine Perino, Anne-Sylvie Fabiano Tixier

**Affiliations:** GREEN Extraction Team, INRA, UMR408, Avignon University, F-84000 Avignon, France

**Keywords:** alternative solvents, green extraction, bio-based solvent, ionic liquids, NADES, water, solvent-free, compressed gas, supercritical solvent, intensification

## Abstract

In recent years, almost all extraction processes in the perfume, cosmetic, pharmaceutical, food ingredients, nutraceuticals, biofuel and fine chemical industries rely massively on solvents, the majority of which have petroleum origins. The intricate processing steps involved in the industrial extraction cycle makes it increasingly difficult to predict the overall environmental impact; despite the tremendous energy consumption and the substantial usage of solvents, often the yields are indicated in decimals. The ideal alternative solvents suitable for green extraction should have high solvency, high flash points with low toxicity and low environmental impacts, be easily biodegradable, obtained from renewable (non-petrochemical) resources at a reasonable price and should be easy to recycle without any deleterious effect to the environment. Finding the perfect solvent that meets all the aforementioned requirements is a challenging task, thus the decision for the optimum solvent will always be a compromise depending on the process, the plant and the target molecules. The objective of this comprehensive review is to furnish a vivid picture of current knowledge on alternative, green solvents used in laboratories and industries alike for the extraction of natural products focusing on original methods, innovation, protocols, and development of safe products.

## 1. Introduction

“*What you see is what you extract*”, with this sentence Choi and Verpoorte [1] pointed that solvent extraction is one of the most important steps in sample preparation for phytochemical analysis but we can also generalize to industrial production via extraction of aromas, colors, antioxidants, fat and oils and fine chemicals for food, cosmetic, perfumery, and pharmaceutical industries. Extraction solvents are principally volatile organic compounds obtained from non-renewable resources, mainly petroleum-based, and suspected to be harmful to both human health and the environment. One such voluminously used solvent is *n*-hexane, a product of controlled fractional distillation from petroleum mixtures. The primary advantage of such solvents are the ease of production, and the chemical properties it possesses that impart ideal functionalities, particularly in terms of solubility for a variety of products, including vegetable oils. Nevertheless, hexane is produced from fossil sources and has recently been classified as CMR 3, which means that it is a suspected reprotoxic category 2 substance under the European Directives and Registration, Evaluation, Authorization and Restriction of Chemicals (REACH) regulations. Due to the new emphasis on environmental and safety protections and the development of green chemistry, finding alternative solvents to petroleum-derived solvents has become a major concern for chemists [2].

This review articulates the current knowledge on alternative, green solvents used in laboratories and industries alike for the extraction of natural products focusing on original methods, innovation, protocols, and development of safe products. It should be noted that it does not automatically imply complete disposal of all the hazards and issues linked with process implementation as new process modification involves automatically new risks. This review aims to be a complete perspective but will not systematically address the following topics, which were pertinently covered by recent or well-established reviews:

- Solvent selection guides offering clear directives and technical data, extensively presented by pharmaceutical companies: GSK [3], Pfizer [4], and Sanofi [5].
- Evaluation tools of interaction between solvent and solute and their chemical properties such as Hansen [6] and Cosmo [7].
- Reverse engineering selection for a new platform of solvents [8].
- Life Cycle Analysis of petroleum versus green and alternative solvents [2].

## 2. Solvent-Free Extraction

As Kerton and Mariotte [2] pointed out with their statement “*the greenest solvent, in terms of reducing waste, is no solvent*”, we should always ask ourselves if we really need a solvent. One of the most famous solvent-free extractions dating back to antiquity is the extraction of olive oil by mechanical pressing. Olive oils are extracted from the fruits of the olive tree using only physical actions, including crushing of olive fruits, and mixing and separation of the olive oil from the resulting paste. This technique has many advantages such as co-extraction of lipophilic and hydrophilic compounds, lipids with natural antioxidants that inhibit lipid autoxidation, but also a large number of volatile and non-volatile compounds responsible for aroma and taste. In the 18th century, cold pressing was also used for extraction to obtain essential oils, or more precisely essences, present in the peels of citrus fruits. Cold pressing or expression is a technique that originated in Sicily and Calabria, before being used by all citrus-growing countries. It consists in diluting the pericarp or “zests” (also called flavedo) so that the essence contained in the oil bags, which line the peel of the fruit, flows outside to be recovered by some device.

In recent years, development of solvent-free techniques appeared to be of great interest in order to modernize conventional processes based nowadays on petroleum solvent extraction. The positive features of solvent-free extraction are numerous: (i) reducing the costs and risks associated with the use of organic solvents; (ii) facilitating scale-up; (iii) enhancing safety by reducing the risk of overpressure and explosion. Several techniques such as instantaneous controlled pressure drop (DIC), pulsed electric fields (PEF), and microwave irradiation (MW) are used to successfully perform solvent-free extractions of primary and secondary metabolites (essential oils, aromas, edible oils, antioxidants, and other organic compounds). These are innovative techniques that allow extractions to be carried out in a practical and efficient way by reducing the extraction time from a few hours to a few minutes because there is no distillation of the solvent, the limiting step of the processes. They eliminate post-treatment of wastewater and normally consume only a fraction of the energy used in a conventional petroleum solvent extraction method.

**Figure 1:**
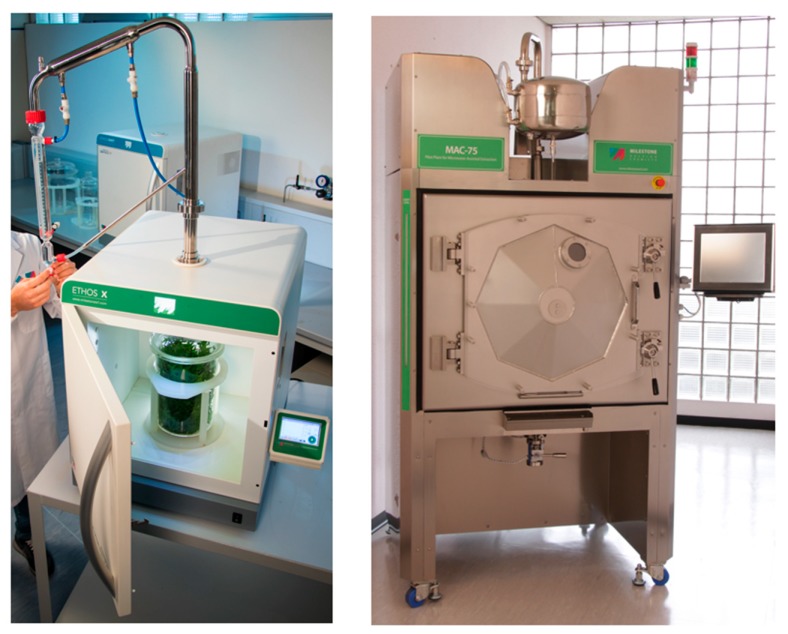
Microwave solvent-free extraction: From analytical lab to industrial scale.

For example, solvent-free microwave extraction (SFME) uses fresh plant materials without addition of any solvent. The principle of extraction is as follows: water plant cells are stimulated by internal heating produced under microwave irradiation, so immediate heating results in a subsequent pressure and temperature increase inside the plant cell, which distends the cell walls and leads to their eventual breakdown and the release of target cellular contents (Figure 1) [9].

The SFME technique has been modified to an original “upside down” alembic called Microwave Hydrodiffusion and Gravity (MHG) [10]. Fresh plants are introduced into the reactor without the addition of solvents. The physical hydrodiffusion phenomenon allows the extracts to drop out of the MW reactor under the effect of gravity. Microwaves heat the plant’s internal water which causes the rupture of cell walls and all the possible metabolites, including the internal water of the plant, will be released and transferred from inside to outside the plant. Solvent Free Extraction techniques like SMFE or MHG have been applied to a variety of fresh aromatic plants, citrus, onions and fruit by-products as presented in Table 1.

With SFME, in most cases, the isolated essential oils contain a substantially lower amounts of monoterpene hydrocarbons and higher amounts of oxygenated monoterpenes than those extracted by conventional methods. MHG is used to extract many compounds of interest such as colors, antioxidants, antimicrobials, etc. These solvent-free extraction methods are much faster, simplified, eco-friendly procedures with high efficiency that allow extraction of bioactive components with reduced energy.

## 3. Water as Green Solvent

From a “natural” viewpoint, water appears as the greenest solvent. It is not only inexpensive and environmentally benign, but is also non-toxic, non-flammable, providing opportunities for clean processing and pollution prevention. The molecule of water is very small with a hard sphere diameter of 2.75 Å. The size of the water molecule, particularly its smallness, is of paramount importance for the hydration of solutes. The two partial positive charges on the hydrogen [H^+^] atoms and the only single zone of negative charge on the oxygen [O^−^] atom gives the water molecule a dipole moment of 1.85D. Whilst considering the contours of the total electron density of the water molecule in the HOH plane the shape it resembles is a spherical one. One among the most important parameters considered for characterizing the polarity of the medium and the control exerted over the ionic dissociation of salts is the macroscopic dielectric constant of a solvent (εr). The high polarity of water can be attributed to dipole orientations of the hydrogen-bond network present giving it a dielectric constant value of 78.3. At higher temperatures and pressures the polarity of water is significantly reduced as the hydrogen bond network is disintegrated. Water has been used for extraction of food and natural products for centuries with different processes and procedures: maceration, decoction, infusion, and percolation, but it is known to be a bad solvent for non-polar or some semi-polar compounds.

The use of enzymes provides high selectivity, mild treatment conditions (processes occurring at low temperatures and for short period time), the possibility of using the whole plant material and high product quality (low residue levels) compared with other methods such as mechanical and chemical treatment [26,27]. For example, Kahveci et al. [28] used enzyme-assisted extraction to increase the recovery of carotenoids, especially lycopene, from tomato paste production waste. A study was carried out on different types of enzymes (proteases and glucanases) and operations conditions *(*5 < pH<9; 30 °C<temperature<50  °C)  to extract the bioactive components on spirulina oil. *Spirulina* oils obtained by enzyme-assisted extraction were richer in essential fatty acids such as palmitic, linolenic and linoleic [29].

**Figure 2:**
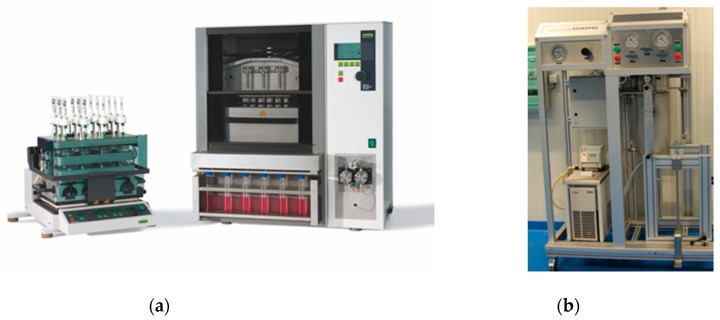
Subcritical water extraction system. (**a**) laboratory scale (www.buchi.com). (**b**) pilot scale (www.zippertex.com).

Subcritical Water Extraction (SWE) uses water under quite different pressure and temperature conditions and therefore needs specific equipment. Subcritical water ocurrs at temperatures between the boiling point and critical point of water (100 °C at 1 bar and 374 °C at 221 bar), at pressures high enough to keep water in a liquid state. It was observed that temperature has a greater effect than the pressure on the polarity of subcritical water. Ideally, the organic molecules are more soluble in water under subcritical conditions due to the lower polarity exhibited it such a state. The dielectric constant is the parameter used to calculate the polarity of water under subcritical conditions. When water is heated above 100 °C the dielectric constant decreases and water tends to exhibit properties similar to those of organic solvents [30]. At 214 °C, the dielectric constant of water is the same as that of methanol at room temperature. At 295 °C water becomes similar to acetone. For this reason, it is possible to extract non-polar, moderately polar, and polar chemical compounds. Low-temperature water extraction could obtain more water-soluble substances, while high-temperature water extraction could extract less soluble substances. Furthermore, liquid water at elevated temperature is a solvent of lower polarizability/polarity. Above 200 °C water may be an acid or base catalyst because its H_3_O^+^ and OH^−^ ions concentrations are perhaps orders of magnitude higher than in ambient water. Subcritical water is, therefore, a much better solvent for hydrophobic organic molecules than ambient water. It can itself be a catalyst for reactions which normally require an added acid or base. Subcritical water can be used at several stages of analysis, for example, for the extraction (Figure 2) and subsequent chromatographic separation of analytes.

SWE gained popularity and there are more uses such as extraction of flavor and fragrance compounds, essential oils, fatty acids, carotenoids and phenolic compounds [31,32,33,34]. For example, Lachos-Perez et al. [35] have used SWE by varying the temperature-dependent dielectric constant to extract flavanones such as hesperidin and narirutin from defatted orange peel. Their experiments were performed in a semi-continuous flow extractor (i.e., batch for the solid and continuous for the liquid). The maximum yields of hesperidin and narirutin were obtained at 150 °C and 10 mL/min. These yields accounted for approximately 21% of the total amount of these flavanones in the extracts, leading to the purest extracts obtained in SWE.

Micellar-assisted extraction using micellar media as the extractant is based on forming micelles by introducing suitable surfactants into an aqueous solution. Surfactants are amphiphilic molecules with a hydrophilic head and a hydrophobic tail. They exist in different shapes and have varying electrical properties: nonionic, cationic, and zwitterionic. When the concentration of surfactant is equivalent to/or exceeds its critical micellar concentration (CMC), the surfactant molecules self assembles, leading to the formation of micelles. The concentration and nature of the surfactant molecules heavily influence the structure of the micelles, thus paving the way for different geometries. For instance, the simplest micelle in water adopts the shape of a sphere bordered by surfactant molecules. These structures are thermodynamically stable under defined conditions depending on the nature of the surfactant, pH, temperature and other solutes. Micellar extraction offers a convenient alternative to conventional extraction systems since it is a fast, highly efficient, simple and user-friendly technique. Su et al. [36] combined a surfactant (Tween-80) and a microwave- assisted process to extract pectin from orange peel. Pectin is commonly extracted from apple pomace or citrus peel using hot water for several hours and at acid pH. Under optimal microwave conditions and in the presence of surfactant, the pectin yield is increased by 17% compared with microwave- assisted extraction only.

While micellar extraction is similar to liquid-liquid extraction, hydrotropic extraction only involves one continuous liquid phase. This method uses amphiphilic organic substances with a short alkyl chain or an aromatic ring with a short alkyl chain, attached to a strongly polar/ionic group, called hydrotropes. Hydrotropy is the phenomena of increasing the solubility of hydrophobic molecules in water by the addition of water-soluble organic molecules. They were first described by Neuberg [37] in 1916 as organic salts able to increase the solubility in the aqueous solutions of an organic compound with low solubility. Hydrotropes may not form micelles (unlike surfactants). The difference between surfactants and hydrotrope is given by the concentration used. For the surfactants, the concentration is of the order of millimolar or less (CMC) while for hydrotropes is in the molar range (the minimum hydrotrope concentration, MHC) which makes the mass of hydrotropes in water tens or hundreds of grams per liter [38]. Consequently, the surface tension decrease of hydrotrope solutions requires these higher concentrations. Hydrotrope-assisted extraction is done through the aggregation of hydrotropes around hydrophobic molecules. Dandekar et al. [39] proposed a new process for limonoid aglycone extraction using aqueous hydrotropic solutions. Two different hydrotropes, sodium salicylate (Na-Sal) and sodium cumene sulphonate (Na-CuS) were studied for maximum yield and the reduction of organic solvents. They concluded that the extraction efficiency depended on the hydrotrope concentration, extraction temperature and percentage of raw material loaded. Limonin yield of 0.65 mg/g seeds was reported using NA-CuS as solvent mixture [40].

## 4. Green Solvents from Ionic Liquids (ILs) and Deep Eutectic Solvents (DESs) to Natural Deep Eutectic Solvents (NADESs)

The aversion towards green solvents has generated great interest and growing demand for ionic liquids (ILs), as an alternative to organic solvents which have several major disadvantages such as their high volatility, flammability, and toxicity [41]. ILs could be borderline in term of green solvents, where the heat of combustion for a variety of ILs was established based on literature data, existing correlation values and a purpose-built model was proposed. The study depicts a clear picture of validation models for the fire safety issues pertaining to the utilization of ILs for a plethora of applications. An innovative “safety by design” approach was articulated wherein the magnitude of the harmfulness of ILs were reduced and validated by a combined OECD *Daphnia magna* standardized test and fish immunomarkers assay. Similarly, a multiscale combined experimental approach was considered to provide advanced knowledge about the thermal and combustion hazard profiles of ionic liquids. Altogether, these validation systems and experimental models aim in characterizing the comprehensive physicochemical hazard profiles of ionic liquids.

**Figure 3:**
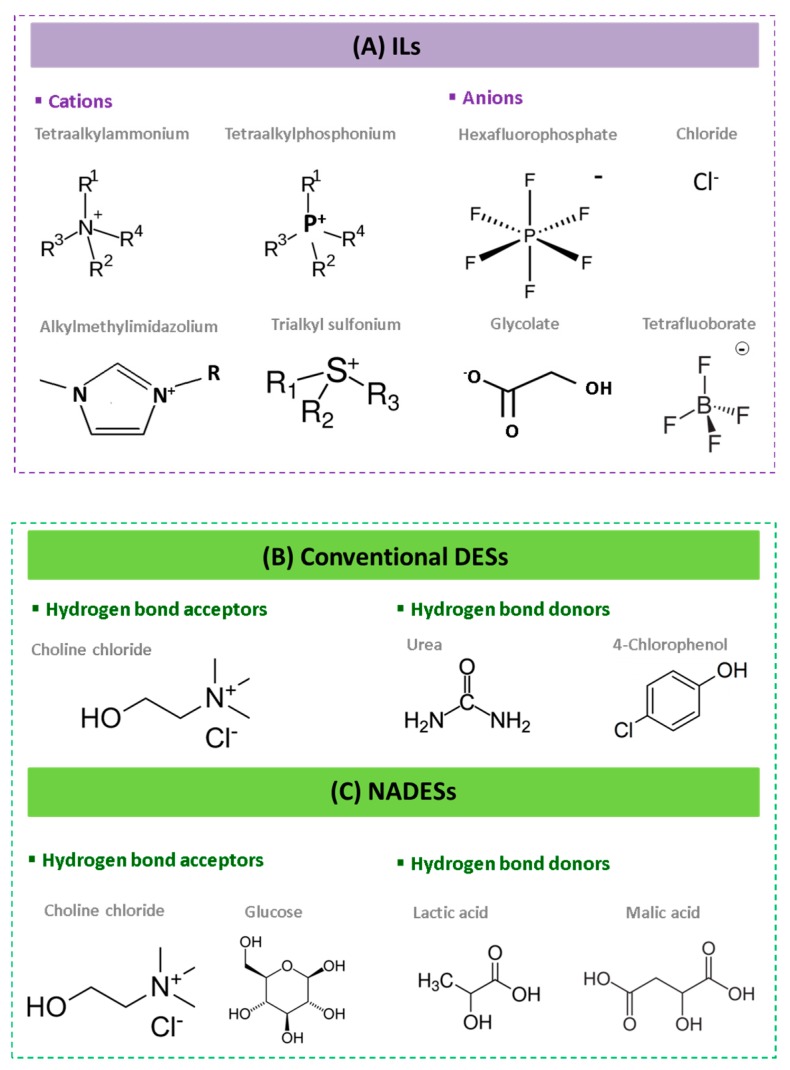
Most common components of ILs, DESs and NADESs. (**A**) Cations and anions most commonly used for the preparation of ILs. Hydrogen bond acceptors and hydrogen bond donors most commonly used for the preparation of DESs (**B**) and NADESs (**C**).

ILs are commonly defined as a group of non-molecular solvents prepared by the combination of organic cations and organic or inorganic anions which melt below 100 °C [42]. The cations and anions most commonly used to prepare ILs are presented in Figure 3.

Among ILs some properties of interest are their non-inflammability, thermal stability, low vapor pressure, and especially their impressive tunability and synthetic versatility [41]. These solvents have long been recognized as green “designer” solvents. Nevertheless, during the past years, their “green” aspect has been widely challenged due to their poor biocompatibility and biodegradability [43,44]. To circumvent this problem, Deep Eutectic Solvents (DESs) have been slowly emerging since 2004, as a green alternative to ILs [45]. DESs, commonly defined as a subclass of ILs, can be prepared by mixing solid compounds which form a eutectic mixture with a melting point lower than either of the individual components melting points [46].

This is mainly due to the generation of intermolecular hydrogen bonds between hydrogen bond acceptor (HBA) and hydrogen bond donor (HBD). DESs share many physicochemical properties with ILs (high viscosity, low volatility, non-inflammability, chemical and thermal stability) [46]. Moreover, they present some advantages over ILs, mainly the ease of their storage and synthesis as well as the low cost of their starting materials [46].

To further meet the principles of green chemistry proposed by Anastas and Warner [47], natural sources of DESs have attracted great attention as replacements of synthetic compounds [48] giving rise to a new class of DESs, namely Natural Deep Eutectic Solvents (NADESs). As is the case of DESs, NADESs are mixtures of compounds that have a much lower melting point than that of any of their individual components [49]. HBA and HBD most commonly used in the preparation of DESs and NADESs are presented in Figure 3.

Besides all the advantages of DESs, NADESs are considered as environmentally friendly and ‘readily biodegradable’ due to the natural origin of their components [45,49], and consequently the obtained extracts can be safely used in the food, pharmaceutical and cosmetics industries [50]. These new green solvents were firstly introduced by Choi and coworkers who defined them as the third liquid phase naturally occurring in all living organisms and cells [51]. According to Choi [51], this third liquid is capable of dissolving a number of natural molecules that are poorly soluble in water and lipids such as taxol and rutin as well as proteins, explaining thus many biological phenomena such as the biosynthesis of molecules that are soluble in neither water nor lipids [51]. The compounds found to form this liquid phase are primary metabolites like organic acids (lactic, malic, citric acids, etc.), sugars (glucose, fructose, sucrose, etc.); amino acids, choline chloride, etc. [44,52]. These natural compounds play key roles in biological processes such as drought resistance, cryoprotection and defense against external attacks [53,54]. Noteworthy, according to the nature of their components, NADESs can be classified into four groups: (1) derivatives of organic acids, (2) derivatives of choline chloride, (3) mixtures of sugars and (4) other combinations [55].

Table 2 summarizes the main properties of ILs and DESs including NADESs. It can be seen that the main difference between ILs and DESs (including NADESs) is the intermolecular force which is based on ionic bonding in the case of ILs and on hydrogen bonding in the case of DESs and NADESs. The other key point is that, contrarily to ILs, DESs and NADESs are non-toxic.

ILs were first observed by Walden in 1914 in the case of ethyl ammonium nitrate [EtNH_3_][NO_3_], which was obtained through the neutralization of ethylamine with concentrated nitric acid [56]. Firstly, they were mainly used for organic synthesis. It was only later that they were explored as extraction solvents. Owing to their high dissolving power and their physicochemical properties tunability, ILs were used as new media in a wide variety of applications. In the last two decades, the literature has revealed several applications of ILs in the extraction of natural products such as plant-based products of high interest for food, nutraceutical and pharmaceutical industries. Throughout the literature, at a laboratory scale, ILs were shown to be more efficient than COS, leading to higher yields of targeted compounds.

Table 3 provides some examples of recent applications of ILs for extraction processes. ILs were applied to extract a large variety of natural products such as phenolic compounds, anthraquinones, tannin, alcohols and essential oils from different plant matrices. Moreover, those alternative solvents were successfully combined with green extraction processes such as UAE and MAE.

A new family of deep eutectic designer solvents were synthesized and formed by crown ether (CE) complexes as HBA and polyethylene glycol (PEG) as HBD. The designer solvents unlock the potential for numerous application in chemistry and material science especially the ultra-deep extraction of non-basic *N*-compounds from fuel oils. The efficacy of p-toluenesulfonic acid-based DES for the extraction of bioactive compounds from Lycium barbarum L. was reported. A 1:2 molar ratio mixture of choline chloride and p-toluenesulfonic acid proved to be the best solvent system for bioactive constituents’ recovery under ultrasound-assisted extraction conditions. The authors attributed the higher extraction efficacy to the solvent systems higher polarity and lower viscosity potential when compared to other DES considered for extraction. Furthermore, DES as a novel extraction media for phenolic compounds from model (safflower) oil using ultrasound wave-assisted liquid-phase microextraction (LPME) method was described. The application of ultrasound aided in extraction time reduction than in the conventional LPME technique.

Despite the high dissolving power of ILs, their toxicity represents a limiting factor of their valorization for both pharmaceutical and food industries, which are subject to strict regulation. Indeed, their toxicological profile was highly questioned in the last decade promoting thus the use of DESs as greener alternatives with great promises in the extraction field. These solvents were firstly observed by Abbott and coworkers in 2003 [68] in the case of choline chloride and urea (ChCl:U) mixture at a molar ratio of (1:2).

Recent studies further proved DESs efficiency in the extraction of natural products and the valorization of food industry by-products, as presented in Table 4. Phenolic acids, flavonoids, volatile compounds as well as primary metabolites (sugars, proteins, etc.) were successfully extracted using DESs. These solvents were proved to provide extracts with higher yields of these compounds of interest in a shorter amount of time, compared to COS. Besides their high viscosity, the synthetic origin of DESs constitutes a major drawback limiting their field of applications.

This is the reason that NADESs are presently getting great attention due to their natural composition [45,49]. Aside from their naturalness, NADESs high stabilization and solubilization abilities make them excellent candidates to replace COS [69]. These natural solvents have been shown to be more efficient than organic solvents in the extraction of plants metabolites and food products and by-products of different polarities while ensuring inexpensive costs and easy preparation methods [43,52]. Moreover, NADESs are less volatile than COS, which guarantees more safety for manipulators [70]. Today, the spectrum of NADESs applications is very broad as shown in Table 4. Flavonoids, phenolic acids, alkaloids, natural pigments, sugars, peptides and volatile compounds are some examples of bioactive compounds that have been successfully extracted from natural matrices using NADESs mixtures. Here, it is worth mentioning that, as the definition of NADESs is recent, some mixtures were reported in scientific publications as DESs while they perfectly fulfill the definition of NADESs.

Owing to their environmental and economic advantages, NADESs appear to be the most promising alternative solvents compared to DESs and ILs. This is the reason that they are presently attracting more attention from industrials. In recent years, an increasing number of companies start to use this natural alternative. Naturex (Avignon, France) is a company specialized in natural ingredients. In 2016, inspired by nature, Naturex patented a new extraction process [92] for the extraction of plant-based active compounds. This is based on the phenomenon of “eutectigenesis” which mimics the intracellular environment. Different mixtures of pure molecules were explored such as (betaine:citric acid) (2:3), (betaine:glycerol) (2:3) and (betaine:lactic acid) (2:3), each containing 25% of water (*w*/*w*) to develop a new range of cosmetic products named “Eutectys™”. These new products were proved to have superior phytochemical profiles and higher biological activities (antioxidant, anti-inflammatory, protection against photo-ageing, etc.) compared to the corresponding conventional hydro-glycerin extracts [92]. Considered as 100% natural, Eutectys™ extracts are easily biodegradable. Moreover, Eutectys^®^ extracts are completely safe as evidenced by their toxicological profiles established according to European regulation (EC n°1223/2009). BASF beauty care solutions, which is a subsidiary of the German company BASF, is based in Lyon, France. This company is specialized in cosmetic ingredients. In 2018, BASF patented the use of coconut water as a solvent for the extraction of natural ingredients especially plant-based compounds. This natural solvent was proved to extract efficiently a wide large of active compounds such as terpenes, flavones, flavonoids, amino acids, lipids, etc. [93].

## 5. Biobased-Solvents

**Figure 4:**
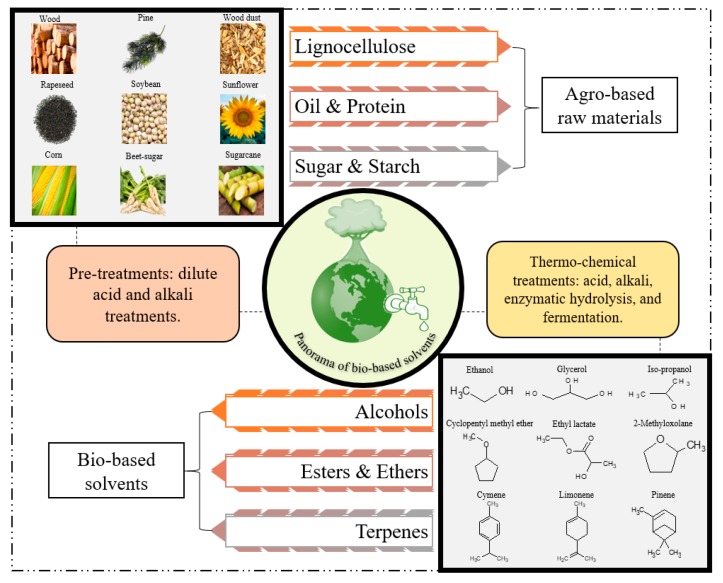
Panorama of bio-based solvents.

Bio-based solvents, as the name signifies, are produced from agricultural biomass. Based on the agricultural origin of the biomass utilized for the production of these solvents they can be predominantly classified into four categories: (a) lignocellulosic; (b) sugar and starch; (c) protein & oil based and (d) other forestry and food wastes. The solvents obtained from these categories can be further classified based on their functional groups (esters, ethers, terpenes, and alcohols) or based on the petroleum-based solvent they were intended to replace (Figure 4). In order to be identified as a green solvent, the solvent should ideally fulfil the twelve criteria proposed in the principles of green chemistry. There are certain parameters and pre-requisites that a typical characteristic solvent should possess to qualify as a green solvent. To name a few, the solvent should be from renewable feedstocks, recyclable using eco-efficient treatments, exhibit similar properties as common solvents, high boiling point and low vapour pressure and enhanced biodegradability under normal environmental conditions, etc. [94,95]. Production or synthesis of bio-based solvents or green solvents it in itself should not have a negative environmental impact.

The agricultural biomass that is used for solvent production has a rigid, fibrous structure as they undergo post-harvest treatments and are secondary intermediates meant for feed purposes or manure. Hence, strong thermo-chemical processes assisted by pretreatments enable the efficient processing of the biomass for suitable solvent production applications. For example, the industrial production of 2-methyltetrahydrofuran (2-MeTHF) involves three steps: (1) an acid treatment of lignocellulosic material to release pentose and hexose sugar units; (2) a biorefining process for the conversion of sugars into furfural and levulinic acid; (3) hydrogenation of levulinic acid with excess hydrogen [96]. Similarly, cyclopentyl methyl ether (CPME) a greener alternative to *tert-*butyl methyl ether (TBME) is synthesized by two methods, firstly a nucleophilic substitution mediated by dimethyl sulfate, where the methylation of cyclopentanol occurs and in the second one where an addition reaction of methanol to cyclopentane is executed (Table 5) [97].

Terpenes (also known as isoprenoids or terpenoids) are mainly acyclic, bicyclic or monocyclic hydrocarbons biosynthetically derived from isoprene units (C_5_H_8_). They exhibit relatively different physical properties and are principally recovered from conifers and fruit pomaces. For instance, limonene is obtained from byproducts of citrus fruit juice production by means of steam distillation and condensation. Microbial production of limonene has immense potential and is of great significance as the bio-conversion of glucose is facilitated by *E. coli* or *S. cerevisiae* and this process avoids the dependency on citrus fruits, and the raw material glucose for fermentation can preferably be sourced from any waste biomass [98].

Ethers like 2-MeTHF and CPME were used as alternative solvents for the extraction of microbial oils, in particular from yeast *Yarrowia lipolytica*, and the oil had similar properties when compared with that of the oil extracted with *n*-hexane [99]. Similarly, the theoretical and experimental solubility of oil obtained from *Pistacia lentiscus* L in various bio-based solvents was elucidated recently, and the in vitro anti-inflammatory activities of the lipid profile was documented [100].

As success stories of the application of bio-based solvents for extraction, an optimized process for lipid extraction with limonene and ethanol as solvent from *Spirulina* microalgae and others using pressurized liquid extraction (PLE) at 200 °C for 15 min was proposed. The extraction procedure had the highest yield for *Spirulina* and the highest amount of ω-3 fatty acids for *Stigeoclonium* [101]. Biodiesel production with dimethyl carbonate (DMC) as a simultaneous extraction solvent and transesterification reagent were explored. Higher yields of fatty acid methyl esters and ethyl esters were reported and a protocol to bypass solvent extraction and oil clean-up using short-chained dialkyl carbonates as a promising method for bio-diesel production was articulated [102]. Three-phase partitioning for the extraction of peroxidase enzyme from a bitter gourd sample by replacing *t*-butanol in the organic phase with DMC is an interesting approach for protein extraction and purification of natural moieties with minimal denaturation [103].

The efficacy of ethyl acetate for the extraction of triterpenoids and polar impurities from birch bark was evaluated, and a higher content of botulin and lower content of polar impurities was obtained when ethyl acetate was employed as extraction solvent. The study also demonstrated the efficient recovery of residual ethyl acetate by hydrodistillation [104]. A microwave-assisted solvent extraction (MASE) system was probed for the extraction optimization of sandbox seed oil. Performance evaluation of three solvent types (acetone, ethyl acetate, and hexane) was compared as a function of oil yield and ethyl acetate proved to be a better solvent with oil yield 1.28 times higher than *n*-hexane under optimized conditions [105]. The stability of curcuminoids, mainly curcumin, demethoxycurcumin, and bisdemethoxycurcumin when ethyl lactate was used in synergy with water (70:30, *v*/*v*) as the extraction system resulted in better stable curcuminoid compounds and its derivatives. Addition of ethyl lactate to the water-based solvent system inhibited the alkaline hydrolysis of the investigated chemical constituents [106]. The potential of PLE with pure ethyl lactate for the removal of caffeine from natural matter, especially green tea leaves with minimized co-extraction of bioactive compounds like catechin, was verified [107]. Such solute-specific elution by ethyl acetate and water solvent systems displays the versatility of bio-based solvents and presents pragmatic solutions for academia and industries alike. Fatty acids were extracted from oilseeds such as soybean, peanuts and sunflower with α-pinene as an alternative to *n*-hexane and the study shed light on the recycling capacity of α-pinene which was closer to 90%, whereas it was a mere 50% for f *n*-hexane, and it was also demonstrated that there was no major degradation in the recycled α-pinene [108]. The suitability of α-pinene as an alternative for the effective replacement of toluene in the moisture determination of food products using Dean-Stark distillation was validated and serves as an example for the incorporation of bio-based solvent for green analytical chemistry [109].

## 6. Liquefied Gases: From Supercritical Fluid to Liquefied Gas Extraction

Supercritical fluids (SCF) are a well-established alternative to traditional organic solvent extraction methods [110]. A fluid reaches its critical state when two phenomena occur simultaneously: (1) when it is heated above its critical temperature (Tc) and (2) when it is pressurized above its critical pressure (Pc). The physicochemical properties of SFE can be manipulated; to obtain specificity in SCF the temperature and pressure can be increased well beyond their critical values. The liquid-like density exhibited by SCF induces a solvating power close to liquids. Their gas-like viscosity results in high mass transfer. Carbon dioxide (CO_2_) is the most widely used supercritical fluid because it is inert, non-toxic, non-inflammable, low cost, abundant, easily removable from the product and possesses moderate critical properties (*T*c = 31.1 °C, *P*c = 7.38 MPa). As a function of pressure and temperature, changes in density can permit variable solvating power, allowing for selective extractions. The versatility of CO_2_ as SCF is well documented, due to its volatility at atmospheric pressure the extracts are solvent-free post depressurization. Considering the fact that supercritical CO_2_ is a non-polar solvent, its solvent power is said to lay between those of pentane and toluene [111]. Usually, a polar co-solvent like methanol or ethanol can be added to enhance the solubilization of polar substances. Carbon dioxide is a generally recognized as safe (GRAS) solvent so products containing extracts obtained with “food grade” carbon dioxide are safe with respect to human health. Many studies have been performed on natural product extraction using supercritical CO_2_, but the high working pressure (*P*c = 7.38 MPa) has limited the industrial applications

In the last decades, the search for new solvents has revived interest in the use of liquefied gases as extraction solvents. Several liquefied gases at a lower pressure (200–1000 kPa), have been used in extraction processes such as *n*-propane, *n*-butane and dimethyl ether. These gases require relatively a very low pressure (<1 MPa) to remain in a liquid state and also they can be easily evaporated at lower temperatures by altering the pressure. Liquified Gas Extraction (LGE) is generally carried out room temperatures with minimal energy consumption and negligible residual solvent in the extracts, thereby preserving the quality of both raw materials and extract. Moreover, existing toxic solvents can be replaced as the chemical structure of LGE makes it suitable for the extraction of lipophilic compounds hence establishing itself as a potential alternative technology within the principles of green extraction of natural products.

For supercritical CO_2_ as an alternative solvent, the extraction process occurs in four stages; the diffusion of the supercritical fluid into the porous sample matrix, the separation of the solute-solute interaction within the matrix, the diffusion of the solutes out of the matrix, and the recovery of the analytes from the sample during decompression.

**Figure 5:**
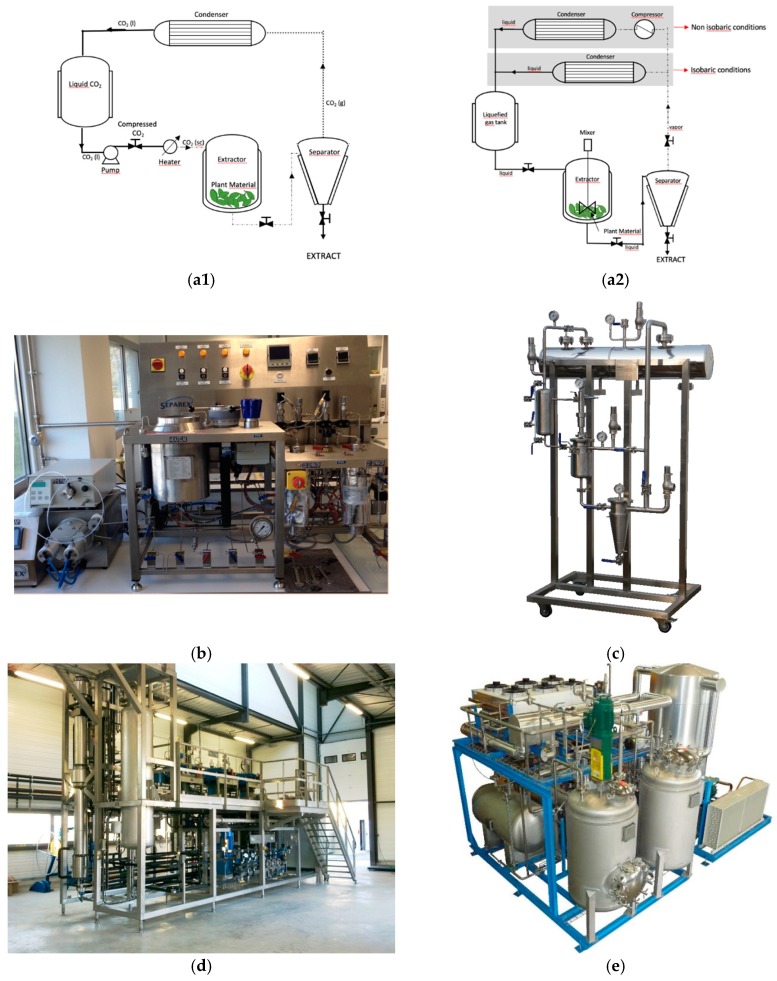
Simplified schematic representation of lab- and pilot-scale unit for extractions using liquefied gases or CO_2_ as a solvent. (**a**) Process diagram of a unit designed for extractions using CO_2_ (**a1**) or liquefied gases (**a2**) as a solvent. (**b**) Example of supercritical CO_2_ lab scale equipment. (**c**) Example of supercritical CO_2_ industrial scale equipment. (**d**) Nectacel 1-L liquefied gas extraction unit manufactured by Celsius Sarl (Villette de Vienne, France). (**e**) 500-L NECTACEL be consistent with names liquefied gas extraction unit manufactured by Celsius Sarl.

Autoclaves are used in supercritical fluid extraction (SFE), and comprise four main components: (i) a pump, to ensure volumetric flow of the fluid; optionally it can be preceded by a cooler for transportation of gaseous components in liquid state, (ii) a heat exchanger, (iii) an extractor, where static and dynamic extractions take place by modulating the pressure regulated by a valve, and (iv) a separator (Figure 5).

Extraction and separation of solute from the solvent are the two main steps involved in SFE. The integral part to perform SFE is to bring the fluid to its supercritical state, this is achieved by sequentially pressurizing and heating the fluid before it enters the extractor. At optimal pressure and temperature, the fluid percolates in the extractor generating ascending or descending flux. Thus, the fluid extracts the solutes present in the matrix. Separation of solute is observed in the separator, where the supercritical fluid returns to its gaseous state and the solutes are separated by gravity. Extracts are collected at the bottom of the separator. The exhaust gas can be recycled by reinjecting it into the system or released into the atmosphere depending on the equipment and processing conditions.

SFE has several key advantages when compared to conventional extraction, chiefly the absence or limited solvent consumption (in case of co-solvent) to produce solvent-free extracts. The number of unit operations is reduced as there is no separation or purification step necessary and the final extract is obtained in the depressurization step. SFE is well suited for heat-sensitive, thermolabile biomolecules as the operating conditions are typically set at lower temperatures.

LGE can be carried out using two mains ways: batch mode or semi-continuous mode. In the beginning, LGE was performed with the conventional way of doing solid-extraction—soaking a matrix in a volume of solvent—but the scientific community quickly realized that semi-continuous modes were more adaptable to those easy-to-evaporate solvents. In the semi-continuous extraction processes, the solvent is continuously evaporated and recycled. In theory, a limited amount of solvent can be used indefinitely until the plant material is exhausted. As semi-continuous processes are not equilibrium limited, even solvents with relatively poor partitioning coefficients but high selectivity can be used with high efficiency. According to the way the solvent flows, semi-extraction processes can be divided into two modes: isobaric and non-isobaric (pressure driven) (Figure 5).

- Non-isobaric: In non-isobaric conditions, the liquefied gas is flowed through the raw material using a circulating pump, then evaporated by expansion and finally liquefied by a compressor. This way of doing is very similar to the working principle of cooling units. Such equipment allows precise control of the flow rate and working pressure. In addition, the solvent can be driven “up-flow” to ensure a maximum solid/liquid contact. However, pumps and compressors are expensive equipment that requires frequent maintenance operations, especially with liquefied gases. Moreover, the size of compressors is typically a limiting factor for large industrial applications, especially in the case of flammable gases.
- Isobaric: Recirculation of solvent can also be achieved without a pump or compressor by using isobaric conditions. In this case, the system always stays at liquid/vapor equilibrium, the operating pressure is equal to the vapor pressure of the solvent. In that case, the liquefied solvent is transferred from a vessel to another by the only help of gravity. The liquefied gas is then evaporated in the boiler under the same pressure (isobaric mode) and the vapors naturally rise to the condenser for solvent regeneration. The absence of mechanical equipment leads to lower energy consumption and maintenance cost. However, the flow-rate only depends on the performance of the boiler and condenser that require careful design and monitoring.

A list of several applications carried out with SFE-CO_2_and LGE such as propane, *n*-butane or DME is presented in Table 6. Extraction by SFE with CO_2_ (SFE-CO_2_) is a well-known technique in both academic institutions and industrial-scale operations and therefore is the subject of numerous research articles and publications. Large scale SFE has been widely used since the late 1970s for the decaffeination of coffee and tea. Applications of SFE-CO_2_ for extraction of natural substances such as oils and fats, flavor and fragrances and pigments from various plants, microorganisms or by-products will continue to be an important research area. SFE-CO_2_ extraction of natural substances is described in several reviews [112,113,114,115,116,117].

Essential oils (EOs) were traditionally extracted from seeds, roots, flowers and leaves using hydrodistillation. Thermal degradation, hydrolysis and solubility of some chemical constituents in water may alter the flavour of the compounds, so SFE-CO_2_ technique can avoid these problems [118,119,120,121]. The optimum operating conditions for extraction of EOs by SFE-CO_2_ method are pressure in the range of 90–250 bar and temperature ranges from 40–50 °C.

For example, Conde-Hernandez and al. [122] published SFE-CO_2_ technique for the isolation of EO from rosemary. Two temperatures (40 and 50 °C) and two pressures (10.34 and 17.24 MPa) were tested and the maximum of EO recovery was between 1.41 and 2.53 g essential oil (EO) 100 g^−1^ of dry rosemary (% *w*/*w*).

Vági et al. [123] compared the extracts produced from the extraction of marjoram (*Origanum maorana* L.) using supercritical CO_2_ (50 °C and 45 MPa) and ethanol Soxhlet extraction. Extraction yields were 3.8 and 9.1%, respectively. Nevertheless, the supercritical extract comprised 21% of essential oil, while the alcoholic extract contained only 9% of the volatile oil substances.

Oil extraction is generally accomplished using hexane which is toxic. It is produced from fossil sources and *n*-hexane, which is one of the main constituents of industrial hexanes, is suspected to be reprotoxic which makes its use at industrial scale questionable. Since the early 1980s, the use of SFE-CO_2_ in the extraction of fats and oils from various plant or animal sources has been studied extensively [124]. For example, Salgin et al. employed a SFE process for the extraction of jojoba oil and investigated the effect of process parameters such as pressure, temperature and particle size of jojoba seeds, the flow rate of CO_2_ on the efficiency of extraction [125].

Because carotenoids are oxidized easily and are sensitive to light and heat, SFE-CO_2_ is a promising method to recover them from plants instead of organic solvents and hot water [126]. In this way, Lima and al. described the extraction of carotenoids from carrot peels by SFE-CO_2_ utilizing ethanol as co-solvent. According to the validated model, the optimal conditions for maximum mass yield (5.31%) were found at 58.5 °C, 306 bar and 14.3% of ethanol, and at 59.0 °C, 349 bar and 15.5% ethanol for carotenoid recovery (86.1%) [127].

Many experimental studies have been carried out in order to evaluate the potential of LGE. In particular, propane, *n*-butane or DME have been extensively investigated for the extraction of fats and oil, antioxidants and aromas [128,129,130,131,132,133,134,135,136,137,138]. As an example, Zanqui et al. [139] experimented with *n*-propane as a solvent for the extraction of chia (*Salvia hispanica* L.) oil, resulting in good extraction yield (28.16%), similar to the Soxhlet method and SC CO_2_, in only 1 h. Moreover, the oil extracted using *n*-propane showed the best oxidative stability as well as the highest contents in polyunsaturated fatty acids (829 mg/g oil), in particular, omega 3 (628 mg/g oil) and omega 6 (201 mg/g oil). Therefore, liquefied gas extraction using *n*-propane was found to be the most efficient method for the extraction of chia oil.

As a solution, Goto et al. [140] proposed an extraction process using dimethyl ether (DME) directly from wet microalgae. They showed that lipids extraction using DME was just as effective as the Bligh-Dyer method (yield = 40.1%), used as reference. Moreover, because of its unique physicochemical properties, DME can extract lipids from microalgae without any preliminary drying and cell disruption steps. As a result, the number of steps and the energy consumption of the overall process could be considerably reduced.

Bier et al. [136] extracted terpenes from agro-industrial waste using a liquid petroleum gas (LPG), composed of a mixture of isomers of propane and butane. This technique was compared to extractions using the Soxhlet method, with *n*-hexane. In particular, they observed that LPG in batch mode at 35 °C resulted in higher yields (5.36% vs. 3.88% using Soxhlet) and high-quality essential oils, composed mainly of limonene (95.3%), α-pinene (0.4%) and β-pinene (0.2%).

Similarly, Nenov et al. [137] described the extraction of essential oil from Ceylon cinnamon tree (*Cinnamomum verum*) using 1,1,1,2-tetrafluoroethane. The extract was composed mainly of cinnamal (77.3%) and coumarin (4.3%), with physicochemical properties comparable to essential oils described in the literature, obtained using either classical extraction methods or SC CO_2_.

## 7. Intensification as a Key for Industrial Success Stories of Green Solvents

In solid-liquid solvent extraction, the equilibrium between solute concentration within the solid and solvent fraction is a function of temperature, solvent type and compounds characteristics. Transfer modes, such as mass, momentum and heat, are involved in this equilibrium and are usually limiting steps. While most studies focus on chemical aspects, such as solvation, it is important to also consider transfer modes. In fact, solubilization in mainly impacted by the conditions under which the solid-liquid extraction was carried out. Conventional extraction processes are not effective in terms of selectivity, performance, energy, yield and environmental impact. Due to new technologies, intensification presents a solution to overcome these obstacles and to enhance one or more transfer modes. There are several technical tools to intensify existing processes such as pulsed electric fields, microwave, and ultrasounds [141,142,143,144,145]. Recently, Arkopharma Laboratories have studied and developed a new process for the extraction of medicinal plants using ultrasonic cavitation. Their study has shown that ultrasound can have a detexturation effect on the plant matrix thus allowing extraction intensification of mass transfer. Results showed a 73% increase in yield concentration coupled with 25% and 33% decreases in energetic consumption and environmental impact, respectively [146].

**Figure 6:**
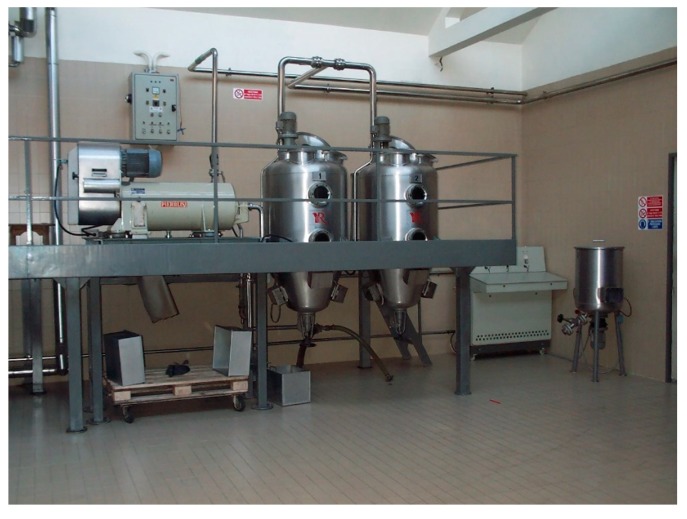
Industrial scale ultrasound extraction process (2 × 500 L) as intensification technique for extraction using vegetable oils (Reus—www.etsreus.com).

Ultrasound as intensification technique has been also used for extraction of food products using vegetable oils as solvents for their aromatization. Veillet et al. [147] proposed an original procedure for the direct aromatization of olive oil with basil leaves using ultrasonic cavitation technique. Another approach was adopted by Li et al. [148] to produce sunflower oil enriched with carotenoids with a reduced number of unit operations compared to the conventional procedure. Yara-Varon et al. [146] present industrial applications of oleo-extracts using vegetable oils as alternative solvents for extraction, purification and formulation (Figure 6).

## 8. Future Trends

Alternative solvents for green extraction of natural products became an innovative research area between industry and academia not only research but also education. It is a new area of innovations that will not end. We will present some future trends which will become industrial success stories in the near future:

● Towards natural ILs

ILs’ applications in the food, nutraceutical and pharmaceutical fields are still limited due to their toxicological profiles. Here, natural sources come into the picture as starting materials for the synthesis of these designer solvents aiming at limiting their toxicity and thus extending their field of applications. Attention has directed towards natural compounds such as lipid compounds [147], amino acids [148] and acids [149,150]. Some anions and cations obtained from natural sources are presented in Table 7.

● Towards new forms of NADESs: THEDESs

A major area of interest would be to further extend NADESs’ field of applications. The pharmaceutical industry is an important field in which NADESs can be of great interest. Therapeutic deep eutectic solvents (THEDESs), defined as bioactive eutectic systems, have been introduced for this purpose. These eutectic mixtures contain an active pharmaceutical ingredient (API) as one of their constituents [157]. THEDESs can be used to enhance the solubility of drugs as well as their permeability, leading to improved pharmaceutical formulations. Only a few researchers have explored these new solvents and proved their positive impacts and their high potential in drugs solubility and permeability [158,159,160,161,162]. Today, THEDESs applications are still limited; but they certainly represent very promising candidates with increasing valorization in the pharmaceutical industry in the near future [163,164,165,166,167,168].

## Figures and Tables

**Table 1:** Solvent free extraction: techniques, applications and experimental conditions.

Material	Analyte	Process/Conditions	Analysis	Ref
Red grape	Anthocyanins	Expeller	UV-visible, HPLC	[11]
Tomato	Carotenoids	Spiral-filter press	UPLC-MS-Ms	[12]
Rice bran	Vegetable oil	Screw press	GC-MS	[13]
Walnut floor	Vegetable oil	Hydraulic press	UV-visible	[14]
Orange peel	Polyphenols	DIC: 0.6 MPa, 20 s, 6 cycles	HPLC-DAD	[15]
Hyssorpus	Essential oil	DIC: 1 MPa, 100 s, 12 cycles	GC-FID, GC-MS	[16]
Roselle	Anthocyanins	DIC: 0.18 MPa, 20 s, 1 cycle	UV-Visible, HPLC	[17]
*Tephrosia* seeds	Ciceritol	DIC: 0.6 MPa, 240 s, 1 cycle	HPLC-DAD	[18]
*Salvia officinalis*	Essential oil	SFME: 650 W, 35 min	GC-MS/GC-FID	[19]
Strawberry	Aromatic compounds	MHG, 1000 W/kg, 30 min.	GC-MS	[20]
Lettuce Onions	Polyphenols	SFME: P.atm, 1 W/g, 15–50 min MHG, 500 g, P(atm) 300–900 W, T = 5–70 min	HPLC-DAD	[21,22]
Tomato	Carotenoids	PEF: 0.5 kV/cm, 1kJ/kg, 60 °C, water	HPLC-DAD	[23]
Purple-fleshed potato	Anthocyanins	PEF: 3.4 kV/cm, 35 pulses, 40 °C, ethanol	HPLC-DAD	[24]
Grape seeds	Polyphenols	PEF: 5 kV/cm, 1–5 pulses, 30% ethanol	UV-Visible	[25]

See text for the corresponding solvent-free technique abbreviations.

**Table 2:** Comparative properties of ILs and DESs including NADESs.

Properties	ILs	DESs Including NADESs
Intermolecular force	Ionic bonding	Hydrogen bonding
Melting point	Below 100 °C	
Vapor pressure	Low	
Viscosity	High viscosity, Positive linear correlation with temperature	
Dissolving ability	A broad range of polar and nonpolar molecules	
Cytotoxicity	Positive for many	Hard to detect

**Table 3:** Recent applications of ILs in extraction procedures.

Material	Method	Analyte	ILs Composition	Ref.
*Ficus carica L.*	UAE	Phenolic compounds	[C_4_MIM][PF_6_](water)	[57]
*Eucalyptus* leaves	MAE		[HO_3_S(CH_2_)_4_MIM]HSO_4_ (water)	[58]
*Lonicerae Japonicae* Flos	UAE		[C_4_MIM]Br (water)	[59]
*Polygonum cuspidatum*	LLE	Polyphenols and anthraquinones	C_6_H_5_Na_3_O_2_ (water); (NH_4_)_2_SO_4_; NaHCO_3_	[60]
Catechu and myrobolan	SPME	Tannin	DIMCARB	[61]
*Suaeda glauca* Bge. Leaves	UAE	Gallic acid	[C_6_MIM]Cl (ethanol)	[62]
*Lotus* leaves	MAE	nornuciferine	[HMIM][Br]	[63]
*Palmarosa* leaves	UAE	Geraniol	DIL-2	[64]
*Farfara*e Flos	Distillation	Essential oils	[C_4_MIM] [CH_3_COO] (water)	[65]
*Spirulina platensis*	UAE	Phycobiliproteins	2-HEAA; [BMIM][Cl]	[66]
*Rehmannia* root	MAE	Verbascoside	[BMIM]Cl	[67]

UAE: Ultrasound-Assisted Extraction; MAE: Microwave-Assisted Extraction; LLE: Liquid-Liquid Extraction; SPME: Solid phase microextrcation; [C_4_MIM][PF_6_]: 1-butyl-3-methylimidazolium hexafluorophosphate; [HO_3_S(CH_2_)_4_MIM]HSO_4_: 3-methyl-1-(4-sulfonylbutyl)imidazolium hydrogen-sulfate; [C_4_MIM]Br: 1-butyl-3-methylimidazolium bromide; DIMCARB: *N*,*N*-dimethylammonium *N*’,*N*’-dimethylcarbamate; [C6MIM]Cl: 1-hexyl-3-methylimidazolium chloride; HMIM][Br]: 1-Butyl-3-methylimidazolium bromide; DIL-2: *N*,*N*,*N*,*N’*,*N’*,*N’*-hexaethylpropane-1,3-diammonium dibromide; [C_4_MIM][CH_3_COO]: 1-butyl-3-methylimidazolium acetate; 2-HEAA: 2-hydroxyethyl-ammonium acetate; 2-HEAF: 2-hydroxyethylammonium formate; [BMIM][Cl]: 1-butyl-3-methyl-imidazolium chloride.

**Table 4:** Recent applications of DESs and NADESs in extraction procedures.

Material	Method	Analyte	DESs/NADESs Composition	Ref.
Grape skin	UAE, MAE	Phenolic Compounds	ChCl:OA, water 25%	[71]
Onion, olive, pear	UAE		LA:Glu; CA:Glu; Fru:CA	[72]
Olive pomace	MAE; UAE		ChCl:CA; ChCl:LA; ChCl:Gly	[73]
Spent coffee	UAE		1,6-HD:ChCl (7:1)	[74]
Orange peel waste	SLE		ChCl:EG (1:4), water 10%	[75]
*Ginkgo biloba*	Stirring	Flavonoids	ChCl:La, water 40% (*w*/*w*)	[76]
PollenTyphae	UAE		ChCl:1,2-PD (1:4), water 30%	[77]
*Radix scutellariae*	UAE		Pro:Gly(1:4)	[78]
*Allium cepa L.*	SLE	Quercetin	ChCl:U	[79]
*Jinqi Jiangtang* Preparations	UAE	Phenolic acids and alkaloids	ChCl:La (1:2); ChCl:Gly (1:2); ChCl:Glu (1:1); Pro:MA (1:1)	[80]
*Chamaecyparis*	HS-SME	Terpenoids	ChCl:EG	[81]
*Artemisia annua*	UAE	Artemisinin	MTA-Ch:B (1:4)	[82]
Shrimp by-products	UAE	Astaxanthin	ChCl:EG; ChCl:Gly; ChCl:1,2-BD; ChCl:1,3-BD; ChCl:1,4-BD	[83]
*Catharanthus roseus*	Heating and stirring	Anthocyanins	ChCl:1,2-PD; LA:Glu; Pro:MA; ChCl:MA; ChCl:Glu; Glu:Fru:Suc	[84]
Wine lees	UAE		ChCl:MA	[85]
Vanilla pods	SLE	Vanillin	14 NADESs/MA:Glu:water (1:1:6); MA:Fru:Glu:water (1:1:1:9)	[52]
*Nicotiana tabacum L.*	MAE	Volatile compounds	ChCl:Gly; ChCl:U; Cap:U	[86]
*Caulis sinomenii*, *Coptis chinensis*, *Stephania tetrandra*, *Sophora flavescens*	UAE	Morphinane, protoberberine, bisbenzylisoquinoline and indole alkaloids	75 types of binary or ternary DESs/ChCl-LA 1:2, 30% water	[87]
Banana puree	MAE	Soluble sugars	MA:BA:water (1:1:3)	[88]
*Averrhoa bilimbi*	Agitation	Pectin	ChCl:CA (1:1)	[89]
Crude palm oil	LLE	Tocols	ChCl:MalA	[90]
Cod skins	Heating and stirring	Collagen peptides	ChCl:U; ChCl:EG; ChCl:Gly; ChCl:LA; ChCl:AA; ChCl:OA	[91]

UAE: Ultrasound-Assisted Extraction; MAE: Microwave-Assisted Extraction; HAE: Homogenate-Assisted Extraction; HHPAE: High hydrostatic Pressure Assisted Extraction; SLE: Solid-Liquid Extraction; LLE: Liquid-Liquid Extraction; ChCl: Choline chloride; OA: Oxalic Acid; LA: Lactic Acid; Glu: Glucose; Fru: Fructose; CA: Citric Acid; 1,6-HD: 1,6-Hexanediol; EG: Ethylene Glycol; La: Laevulinic Acid; 1,2-PD: 1,2-Propanediol; Gly: Glycerol; Pro: Proline; U: Urea; MTA-Ch: Methyl trioctylammonium chloride; B: Butanol; 1,3-BD: 1,3-Butanediol; 1,4-BD: 1,4-Butanediol; 2,3-BD: 2,3-Butanediol; Suc: Sucrose; Cap: Caprolactam; BA: β-Alanine; MalA: Malonic Acid; AA: Acetic Acid.

**Table 5:** Bio-based solvents as an extraction solvent for various analytes.

Analyte	Material	Bio-Based Solvent	Method	Ref.
Oil	*Yarrowia lipolytica*	CPME	Hot reflux	[99]
Oil	*Pistacia Lentiscus* L.	MeTHF	Soxhlet	[100]
Oil	*Anabaena planctonica*	D-limonene	Pressurized liquid extraction	[101]
Oil	*Jatropha curcas* L.	DMC	Maceration	[102]
Peroxidase enzyme	*Momordica charantia*	DMC	Three-phase partitioning	[103]
Triterpenoids	*Betula pendula Roth.*	Ethyl acetate	Reflux	[104]
Oil	*Hura crepitans*	Ethyl acetate	Microwave	[105]
Curcuminoids	*Curcuma longa* L.	Ethyl lactate	Maceration	[106]
Caffeine	*Camellia sinesis*	Ethyl lactate	Pressurized liquid extraction	[107]
Fatty acids	*Arachis Hypogaea*	α-pinene	Soxhlet	[108]

MeTHF—2-methyl tetrahydrofuran; CPME—Cyclopentyl methyl ether; DMC—Dimethyl carbonate.

**Table 6:** Extraction applications with SFE-CO_2_ and LGE as a green solvent.

Material	Analyte	Solvent	T (°C)/P (MPa)	Ref.
*Rosmarinus officinalis*	EO	SFE-CO_2_	40 °C/10.34 MPa; 50 °C/17.24 MPa;	[122]
*Origanum majorana*	EO	SFE-CO_2_	50 °C/45 MPa	[123]
Jojoba seeds	oil	SFE-CO_2_	25–45 °C/67–90 MPa	[125]
*Carapa guianensis*	Fatty acids + phenolic	n-butane	25 °C, 0.7 MP	[128]
Carrot peel	carotenoids	SFE-CO_2_ -Ethanol	58,5 °C/30.6 MPa (with 14.3% of ethanol)	[129]
*Helianthus annuus* L.	Fatty acids	n-butane	40 °C, 0.4 MPa	[129]
*Perilla frutescens*	Lipids	n-propane	40 °C, 0.8 MPa	[131]
*Sesamum indicum* seeds	oil	SFE-CO_2_	19–25 °C/40–60 MPa	[132]
*Sesamum indicum* seeds	Fatty acids + antioxidants + proteins	n-propane	60 °C, 12 MPa	[132]
*Euglena gracilis*	Lipids	DME	20 °C, 0.7 MPa	[133]
*Botryococcus braunii*	Hydrocarbons	DME	20 °C, 0.7 MPa	[134]
*Arthrospira platensis*	lipids	DME	20 °C, 0.5 MPa	[135]
Citrus leaves	Essential oil	DME	35 °C, 0.78 MPa	[135]
Orange Waste	Terpenoids	LPG	35 °C, 0.45 MPa	[136]
*Salvia hispanica* L.	Fatty acids + antioxidants	n-propane	45 °C, 10 MPa	[139]
Microalgae	Lipids	DME	30 °C, 0.7 MPa	[140]

**Table 7:** Anions and cations obtained from natural sources for synthesis of ionic liquids.

Ions	Group	Source	Precursor	Structure	Example of IL	Ref.
**Anions**	Carboxylic acids	Vegetable oils	Oleic acid	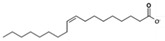	[HE_2_A][C_18_OO]	[151]
	Amino acids	Meat, eggs and dairy foods	Glycine	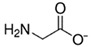	[C_2_mim][Gly]	[152]
			Lysine	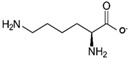	[Ch][Lys]	[153]
**Cations**	Natural amine	Soybeans, eggs and peanuts	choline	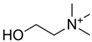	[Ch][Ser]	[154]
					[Ch]Cl	[155]
					[Ch][Ala]	[156]

[HE_2_A][C_18_OO]: Bis(2-hydroxyethyl) ammonium oleate; [C_2_mim][Gly]: 1-ethyl-3-methylimidazolium glycinate; [Ch][Lys]: Cholinium lysine; [Ch][Lys]: Cholinium serine; [Ch]Cl: Cholinium chloride; [Ch][Ala]: Cholinium alanine.
